# Reviewed article: Bicalho JMF, Guimarães AA, Oliveira CDL, Lopes ACS. Perceptions of health professionals about the implementation of the Adequate and Healthy Eating Promotion Program in a municipality of the Minas Gerais: a qualitative study. Epidemiol Serv Saude. 2025:34;e20240408.

**DOI:** 10.1590/S2237-96222025v34e20240408.a

**Published:** 2025-07-11

**Authors:** Italo Wesley Oliveira Aguiar

**Affiliations:** Universidade Federal do Ceara

**Table:** 

Rating	Excellent	Good	Average	Below average	Poor
Interest		✓			
Quality		✓			
Originality				✓	
Overall			✓		

**Table:** 

Questionnaire	Yes	No	Not applicable
Does the manuscript contain new and significant information to justify publication?		✓	
Does the Abstract (Summary) clearly and accurately describe the content of the article?	✓		
Is the problem significant and concisely stated?	✓		
Are the methods described comprehensively?		✓	
Are the interpretations and conclusions justified by the results?	✓		
Is adequate reference made to other work in the field?	✓		

## Manuscript Structure

Length of article is: Adequate

Number of tables is: Too few

Number of figures is: Too few

Please state any conflict(s) of interest that you have in relation to the review of this paper (state “none” if this is not applicable): None

## Comments

Prezados(as) autores(as), parabéns pelo manuscrito, que reporta percepções importantes sobre a intervenção educacional conduzida entre profissionais da saúde. A seguir, apresento alguns comentários com o intuito de contribuir para o aprimoramento do seu trabalho.

Apesar de observar que o seu relato de pesquisa segue uma estrutura suficiente, acredito que ele poderia se beneficiar de uma reorganização segundo diretrizes voltadas para o informe de estudos qualitativos e educacionais, como o SQUIRE-EDU (Standards for QUality Improvement Reporting Excellence in Education) [1] e o SRQR (Standards for Reporting Qualitative Research) [2]. Com relação aos métodos, sugiro que o roteiro utilizado para conduzir a entrevista seja descrito em maiores detalhes e, se possível, adicionado como material suplementar. Isto permitiria que os leitores tivessem uma noção melhor sobre a entrevista que foi realizada.

Na discussão, sugiro adicionar ou explicitar os diálogos realizados entre esta pesquisa e demais estudos distintos, que realizaram intervenções educacionais em amostras de outras localidades em contextos similares. Isto deve-se pelo potencial que este diálogo teria para ajudar a dimensionar a possibilidade de generalizar ou não as percepções em questão. Chama atenção o fato de que o único estado mencionado na discussão seja exatamente aquele onde o estudo foi realizado (Minas Gerais), limitando a oportunidade de comparar e discutir resultados com pesquisas de outras regiões do Brasil. Essa inserção poderia enriquecer a análise, alinhando-se ao alcance nacional e ao escopo da RESS.

Além disso, proponho que os trechos apresentados em “bullets” sejam reescritos, no formato de prosa. Apesar de listagens propiciarem distinção e clareza para os assuntos, o texto contínuo tende a promover uma melhor coesão e fluxo de ideias, permitindo uma argumentação mais elaborada e conectada. Acredito que os “bullets” seriam mais apropriados para slides ou textos informais, voltados a leitores que têm menos disponibilidade de tempo ou menos familiaridade com a temática.

[1] https://pubmed.ncbi.nlm.nih.gov/30998575/

[2] https://pubmed.ncbi.nlm.nih.gov/24979285/

